# Leukoencephalopathy due to inhalational and trans-conjunctival heroin abuse: First case report from Pakistan

**DOI:** 10.1016/j.amsu.2022.103351

**Published:** 2022-02-05

**Authors:** Mohammad Suleman Bajwa, Hassan Nawaz, Muhammad Aemaz Ur Rehman, Sadaf Iftikhar, Abu Hurera

**Affiliations:** Mayo Hospital, King Edward Medical University, Neela Gumbad Chowk Anarkali, Lahore, 54000, Pakistan

**Keywords:** Case report, Heroin, Leukoencephalopathies, Magnetic resonance imaging, Substance-related disorders, White matter

## Abstract

**Introduction:**

and importance: Heroin-induced leukoencephalopathy (HLE) is a rare illness that causes diffuse white matter destruction, leading to acute or subacute development of neurological signs and symptoms. Physicians must be aware of the likely clinical presentation to properly evaluate and diagnose this clinical entity.

**Case presentation:**

We report the case of a young gentleman who presented with acute stupor following his first instance of heroin vapor inhalation. He later confessed to *trans*-conjunctival application of the drug as well. His Glasgow Coma Scale (GCS) score improved within four days of admission, however, the neurologic sequalae such as cognitive impairment, spastic paraparesis and urge incontinence only partially resolved at three months. Abnormal white matter hyperintensities with restricted diffusion on brain magnetic resonance imaging and history of heroin abuse led to diagnosis of toxic leukoencephalopathy.

**Clinical discussion:**

Leukoencephalopathy with heroin is mostly observed after inhalation (i.e., “chasing the dragon”) but other routes of abuse have also been reported. Although a large spectrum of presentations exists, altered mental status, cerebellar dysfunction and fecal/urinary incontinence are the most commonly seen presenting features. Anti-oxidant therapy has shown promising results in terms of treatment.

**Conclusion:**

The growing rates of opioid use disorders require physicians to be aware of and counsel the patients regarding dangerous neurological sequelae of these drugs.

## Highlights

• Heroin-induced leukoencephalopathy (HLE) is a rare entity that can present with neurobehavioral symptoms and focal neurological deficits.
• It is most common after inhalation but *trans*-conjunctival abuse may cause it as well.
• The growing rates heroin abuse require physicians to be aware of and counsel the patients regarding HLE.

## 1 Introduction

Heroin-induced leukoencephalopathy (HLE) is a form of toxic encephalopathy that usually presents with altered consciousness, neurobehavioral symptoms and focal neurologic deficits [1]. It is most commonly reported with inhalational abuse (‘chasing the dragon’), although other routes such as smoking and snorting have also been recognized [[2], [3], [4]]. Wolthers et al., in 1982 described its clinical course in three distinct stages and based on radiological location of lesions, suggested predominant pyramidal and cerebellar symptoms [1]. This is the first reported case of heroin induced encephalopathy from Pakistan and it adds to the growing global literature on this rare clinical entity. A history of tuberculosis, non-conventional mode of abuse (*trans*-conjunctival) and acute leukoencephalopathy following first instance of inhalation are some of the distinctive features of this case. The article also includes a concurrent review of literature to describe the common presentation and underlying pathogenesis of HLE. This case report has been reported in line with the SCARE criteria [5].

## 2 Case report

### 2.1 Presentation and history

A 21-year-old Pakistani man presented to the emergency department in an altered state of consciousness. He had sniffed a drug of abuse 6 hours prior to presentation, developed a high-grade fever responsive to acetaminophen, had two episodes of projectile vomiting and, within an hour of presenting, progressed from irritable to stuporous. Initial examination found the patient febrile and stable, with a GCS of 6/15 (E1M4V1). He had dilated reactive pupils, an upgoing right plantar reflex, and a urine toxicology screen positive for morphine and benzodiazepine. There was no history of preceding falls, headaches, abnormal movements and signs of meningeal irritation were negative.

### 2.2 Differential diagnosis, investigations and treatment

**Fig. 1:**
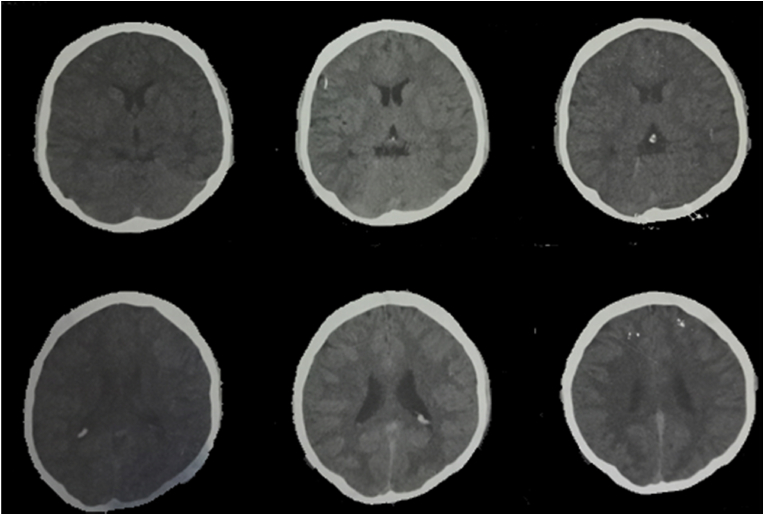
Non-enhanced CT Brain (axial) from the emergency department showing hypo-attenuation of the white matter and diffuse sulcal effacement.

Emergency CT scan (Shown in Fig. 1) revealed diffuse sulcal effacement and hypoattenuation of the white matter. Naloxone was administered emergently; mannitol, dexamethasone, vancomycin, ceftriaxone and acyclovir were started empirically for infectious causes of encephalitis.

Emergency labs revealed leukocytosis (TLC = 16.6 10^9^/L, 82.3% neutrophils), urea 48g/dL, creatinine 1.4g/dL, hyponatremia (Na = 128mEq/L), hyperkalemia (K = 5.8mEq/L). Urine output was approximately 2–2.5 L/day throughout his hospital stay. MRI-contrast studies were delayed till the renal markers normalized. He had a history of pulmonary tuberculosis diagnosed 7 months ago, managed with antituberculosis therapy which ended 1 month prior to admission.

The patient was admitted to the Medical ICU, where he was managed as a case of meningoencephalitis, with differentials of tuberculous meningitis, drug induced encephalopathy, acute disseminated encephalomyelitis (ADEM) and cerebral malaria. CSF analysis was unremarkable, with all microscopic and biochemical parameters, including Adenosine Deaminase (ADA), within normal limits. On the first day of admission, he suffered an isolated episode of generalized tonic-clonic seizure but his GCS improved gradually, reaching 15/15 on the 4th day of admission, after which mannitol was discontinued. Opioid substitution treatment was initiated, with intravenous nalbuphine twice daily, later shifted to oral tramadol with alprazolam and finally to NSAIDs. On regaining consciousness, he complained of spastic paraparesis and urinary incontinence suggestive of spastic bladder.

This was later accompanied by colicky abdominal pain. The abdomen was non-tender and the remaining abdominal exam was unremarkable. Ultrasound revealed a contracted gallbladder full of calculi and bilateral grade-I renal parenchymal disease. Clinical examination and subsequent imaging studies ruled out biliary, intestinal and renal colic and the paroxysmal pain was diagnosed as skeletal muscle spasms. These spasms were not responsive to analgesics.

On the 15th day of admission, he was shifted to the Neurology ward for evaluation. In confidence, the patient admitted to experimenting with drugs of abuse by the conjunctival route, which were associated with red eyes and headaches relieved by aspirin. The abuse that precipitated his clinical condition involved ‘burning a white powder in foil and sniffing the fumes’. Laboratory and clinical examination indicated that the abused substance was a mixture of heroin, benzodiazepines and adulterants. He revealed no other instances of drug abuse.

**Fig. 2:**
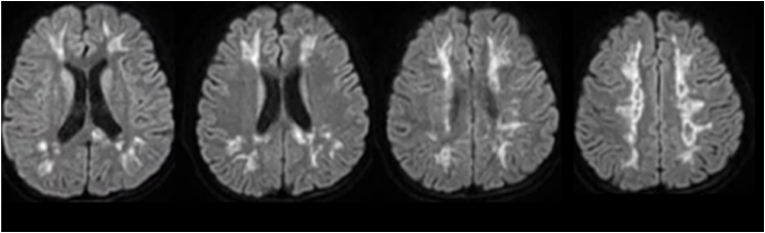
MRI Brain T2-FLAIR (axial) showing white matter hyperintensities in the centrum semiovale, corona radiate and splenium of corpus callosum.

Abnormalities in mentation included poor concentration, decreased awareness of surroundings, delayed response to questions and commands, and poor recall (MMSE = 21). Examination revealed pupils to be dilated and non-reactive, but cranial nerves, sensory and cerebellar systems were found to be normal. Lower limbs showed symmetrical findings of hypotonia, 3/5 power proximally, 2/5 distally, and brisk deep tendon reflexes. Right plantar was upgoing, while the left was mute. MRI findings revealed T2-FLAIR bilateral hyperintensities in deep white matter with diffusion restriction on DWI sequences (Shown in Fig. 2), hence the diagnosis of bilateral drug-induced cerebral leukoencephalopathy was made. The patient could not afford high dose vitamin C or E so coenzyme Q10 and turmeric powder were advised instead.

During his hospital course, we observed certain signs resembling opioid withdrawal. These included diaphoresis, mydriasis, yawning, insomnia, abdominal pain and muscle spasms. The locus ceruleus, whose norepinephrine production is responsible for most features of withdrawal [6], was spared on MRI. As these signs failed to respond to opioid substitution therapy, and widespread leukoencephalopathy was observed, these signs cannot be attributed to uncomplicated opioid withdrawal. Further, his history suggested only three instances of abuse, so dependence may not have fully developed.

### 2.3 Outcome and follow-up

The patient responded to treatment and partial improvement in his cognition, spastic paraparesis and urge incontinence was noted upon follow up at three months.

## 3 Discussion

Heroin induced leukoencephalopathy (HLE) was first described as a separate clinical entity in a cohort of patients from Netherlands [1]. Users heat the heroin powder after placing it on an aluminum foil – the resulting smoke resembles a dragon's tail and the act of sniffing is colloquially known as ‘chasing the dragon’. Although inhalational abuse was previously thought as the only major culprit, newer evidence from case reports has emerged that suggests that other means of administration (i.e., injecting or snorting) are also likely to lead to this clinical entity [7,8]. To our knowledge, this is the first reported case of HLE from Pakistan and it adds to the growing global literature on heroin-induced leukoencephalopathy. The paucity of large-scale clinical data on this topic makes the reporting and dissemination of such cases very important.

HLE is classified as mild, moderate and severe in terms of clinical symptoms, however this delineation is often difficult to appreciate [8]. A review of cases has identified altered mentation, confusion, cerebellar dysfunction, fecal/urinary incontinence as presenting features in most cases of heroin leukoencephalopathy [7]. Corticospinal and extrapyramidal tract involvement leading to weakness and hyperreflexia can also be seen. Our patient also had complaints of mental changes, incontinence, weakness and abnormal reflexes among other findings. Inhalational abuse classically leads to diffuse white matter hyperintensities in cerebellum, posterior cerebrum, posterior limbs of the internal capsule and corpus callosum while other routes of administration may cause symmetric hyperintensities in frontoparietal white matter, centrum semiovale and genu of the internal capsule [7]. Spongiform degeneration of the white matter with no demyelination is the key finding on neuropathology [9].

Although the exact cause is unknown, multiple hypotheses exist in terms of underlying pathogenesis. The chemical change caused by heating forms a compound called heroin pyrolysate, the toxins of which have been implicated in the disease process. Elevated lactate in white matter and the possible response to antioxidants also suggests mitochondrial dysfunction [9]. As the heroin pyrosylate is often prepared on aluminum foil, the possibility of concurrent aluminum leukoencephalopathy exists, but the symptoms of isolated aluminum leukoencephalopathy are found to be strikingly different from heroin leukoencephalopathy [10].

Though this disease lacks a specific treatment, antioxidant therapy holds therapeutic potential^3^. In resource-limited settings, turmeric extract, whose neuroprotective and antioxidant roles are well studied, may have a role in therapy [3]. Although our patient did not recover completely, significant clinical improvement was observed after receiving turmeric and coenzyme Q10.

## 4 Strengths and limitations

Features particular to this case include presentation in a state of stupor with cerebral edema and supratentorial leukoencephalopathy after a single instance of sniffing, with a recent history of its administration via conjunctival route. Our patient also had a recent history of tuberculosis, which has been shown to increase the permeability of blood brain barrier [11]. The increased permeability might have played some role in augmenting the toxic effects of heroin on brain but the absence of ADA on CSF analysis and paucity of literature to support this argument makes it a questionable inference. We acknowledge that this patient received a delayed diagnosis. In addition to the rarity of the syndrome itself, potential reasons for this delay were his presentation closely mimicking alternative diagnoses (that are much more likely to be seen in Pakistan), and unavailability of MRI in the emergency setting. 6-monoacetylmorphine, a metabolite specific for heroin, could not be tested because more than 8 h had elapsed since the patient last used the drug of abuse.

## 5 Conclusion

Leukoencephalopathy is a rare complication of heroin abuse which should be suspected in patients with appropriate history, positive urine toxicology and neurobehavioral symptoms. With rise of drug abuse in developing countries, practicing physicians should be aware of and counsel patients regarding potentially fatal complications like toxic leukoencephalopathy.

## Funding sources

This study did not receive any specific grant from funding agencies in the public, commercial, or not-for-profit sectors.

## Ethical approval

Not applicable.

## Consent

Written informed consent was obtained from the patient for publication of this case report and accompanying images. A copy of the written consent is available for review by the Editor-in-Chief of this journal on request.

## Author contributions

All the authors have met the minimum criteria for authorship and are in agreement regarding the contents of the manuscript. **MSB** managed the patient as a physician and wrote the initial draft. **HN** performed relevant literature search and improved the initial draft. **MAUR** worked on the discussion section and critically reviewed the manuscript for content and clarity. **SI** was involved in the patient management as a neurologist and approved the final submission. **AH** was involved in reviewing the whole manuscript and suggested important revisions.

## Registration of research studies

Name of the registry: Not Applicable.

Unique Identifying number or registration ID: Not Applicable.

Hyperlink to your specific registration (must be publicly accessible and will be checked): Not Applicable.

## Guarantor

I, Muhammad Aemaz Ur Rehman, the corresponding author, accept my role as the Guarantor of this case report.

## Provenance and peer review

Not commissioned, externally peer-reviewed.

## Declaration of competing interest

The authors have no conflict of interest to declare.
